# Supplementary material for: Connexin-43-dependent ATP release mediates macrophage activation during sepsis
Source: eLife. 2019 Feb 8;8:e42670. doi: 10.7554/eLife.42670 (PMC6415938; doi:10.7554/eLife.42670)
Supplement: Supplementary file 1. — (A) Diagnosis of human patients included in the study. (B) P2 receptors antagonists. (C) Visceral Surgery Murine Sepsis Score Sheet. (D) Anti-mouse antibodies used for flow cytometry (E) Anti-human antibodies used for flow cytometry (F) Anti-mouse antibodies used for Time-of-Flight Mass Cytometry [file elife-42670-supp1.docx]

**Supplementary File 1**

**Supplementary file 1A. Diagnosis of human patients included in the study.**

| **N°** | **Gender** | **Age** | **Diagnosis** | **Surgical procedure** | **Elective/**  **Emergency** | **Peritonitis (Yes/No)** |
| --- | --- | --- | --- | --- | --- | --- |
| **1** | Female | 61 | Morbid obesity | Roux-en-Y gastric bypass | Elective | No |
| **2** | Male | 51 | Gallstones | Laparoscopic cholecystectomy | Elective | No |
| **3** | Male | 58 | Symptomatic Gallstones | Laparoscopic cholecystectomy | Elective | No |
| **4** | Female | 73 | Pancreatic adenocarcinoma | Distal pancreatectomy with splenectomy | Elective | No |
| **5** | Female | 71 | Gastroesophageal reflux disease | Laparoscopic fundoplication | Elective | No |
| **6** | Male | 35 | Perforated peptic ulcer | Laparoscopic suture | Emergency | Yes |
| **7** | Male | 47 | Intraperitoneal abscess | Laparoscopy, Ileostomy | Emergency | Yes |
| **8** | Female | 77 | Necrotising pancreatitis | Laparoscopic drainage | Emergency | Yes |
| **9** | Male | 47 | Appendicitis | Laparoscopic appendectomy | Emergency | Yes |
| **10** | Female | 73 | Perforated peptic ulcer | Laparoscopic suture | Emergency | Yes |
| **11** | Male | 34 | Appendicitis | Laparoscopic appendectomy | Emergency | Yes |

**Supplementary file 1B. P2 receptors antagonists.**

| **P2 receptors targeted** | **Compounds** | **Supplier, Reference number** |
| --- | --- | --- |
| Non-selective P2R antagonist | Suramin | Tocris Bioscience, #1472 |
| P2RX3 | NF 110 | Tocris Bioscience, #2548/10 |
| P2RX4 | 5-BDBD | Tocris Bioscience, #3579 |
| P2RX7 | A 804598 | Tocris Bioscience, #4473 |
| P2RY1 | MRS 2279 | Tocris Bioscience, #2158 |
| P2RY2 | A-RC 118925XX | Tocris Bioscience, #4890 |
| P2RY6 | MRS 2578 | Tocris Bioscience, #2146/10 |
| P2RY11/X1 | NF 157 | Tocris Bioscience, #2450/10 |

**Supplementary file 1C. Visceral Surgery Murine Sepsis Score Sheet.**

**Starting body weight (BW):** **Timepoint**

| Variable | **Score and description** | **1** | 2 | 3 | 4 | 5 | 6 | 7 | 8 |
| --- | --- | --- | --- | --- | --- | --- | --- | --- | --- |
|  | Time |  |  |  |  |  |  |  |  |
| Appearance | 0 – Coat is smooth  1 – Patches of hair piloerected  2 – Majority of back is piloerected  3 – Piloerection and mouse appears “puffy” or emaciated |  |  |  |  |  |  |  |  |
| Level of consciousness | 0 – Mouse is active  1 – Mouse is active but avoids standing upright  2 – Mouse activity is noticeably slowed. The mouse is still ambulant, only moves when provoked  3 – Activity severely impaired. Mouse remains stationary when provoked, with possible tremor |  |  |  |  |  |  |  |  |
| Activity | 0 – Normal amount of activity. Mouse is any of: eating, drinking, climbing, running, fighting  1 – Slightly suppressed activity. Mouse is moving around bottom of cage  2 – Suppressed activity. Mouse is stationary with occasional investigative movements.  3 – No activity. Mouse is stationary and/or experiencing tremors, particularly in the hind legs |  |  |  |  |  |  |  |  |
| Response to stimulus | 0 – Mouse responds immediately to auditory stimulus or touch  1 – Slow or no response to auditory stimulus; strong response to touch (moves to escape)  2 – No response to auditory stimulus; moderate response to touch (moves a few steps)  3 – No response to auditory stimulus; little or no response to touch. Cannot right itself if pushed over |  |  |  |  |  |  |  |  |
| Eyes | 0 – Open  1 – Eyes not fully open, possibly with secretions  2 – Eyes at least half closed, possibly with secretions  3 – Eyes half closed to closed, possibly with milky secretions |  |  |  |  |  |  |  |  |
| Respiratory rate | 0 – Normal, rapid mouse respiration  1 – Slightly decreased respiration (rate not quantifiable by eye)  2 – Moderately reduced respiration (rate at the upper range of quantifying by eye)  3 – Severely reduced respiration (rate easily countable by eye, >1 s between breaths) |  |  |  |  |  |  |  |  |
| Respiratory quality | 0 – Normal  1 – Brief periods of laboured breathing  2 – Laboured, no gasping  3 – Laboured with intermittent gasps to consistent gasping |  |  |  |  |  |  |  |  |

**Scoring:**

- Score of 0 requires no action
- Scores of 1 or 2 in any category, requires careful monitoring (Score 1 = 2 x per day; Score 2; every 2 hours) for worsening of symptoms and additional analgesic (dose = 0.05 mg/kg Temgesic^®^, s.c. injection) will be administered.
- Score of 3 in any category, animal will be sacrificed.

**Supplementary file 1D. Anti-mouse antibodies used for flow cytometry**

| **Target** | **Fluorochrome** | **Clone** | **Source** | **Catalogue n°** |
| --- | --- | --- | --- | --- |
| CD11b | APC-Cy7 | M1/70 | Bio Legend | 101226 |
| CD11c | eFluor450 | N418 | eBioscience | 48-0114-82 |
| CD163 | Pacific blue | GHI/61 | Bio Legend | 333611 |
| CD19 | BV570 | 6D5 | Bio Legend | 115535 |
| CD3 | Alexa Fluor 700 | 17A2 | Bio Legend | 100216 |
| CD4 | APC | RM4-4 | Bio Legend | 116014 |
| CD44 | PE-eFluor610 | IM7 | eBioscience | 61-0441-82 |
| CD45 | Alexa Fluor 594 | 30-F11 | eBioscience | 103144 |
| CD45 | BUV395 | 30-F11(Ruo) | BD Biosciences | 564279 |
| CD62L | BV421 | MEL-14 | BD Biosciences | 562910 |
| CD69 | PE-Cy7 | H1.2F3 | BD Biosciences | 552879 |
| CD8a | PE | 53-6.7 | Biolegend | 100708 |
| CX43 | Alexa Fluor 488 | CX-1B1 | Invitrogen | 138388 |
| F4/80 | BUV 395 | T45-2342 | BD Bioscience | 565614 |
| F4/80 | APC | BM8 | EBioscience | 17-4801-82 |
| FoxP3 | Percp cy.5.5 | FJK-16s | eBioscience | 45-5773-80 |
| GATA6 | PE | D61E4 | Cell Signaling | 26452 |
| Ly6C | APC | HK1.4 | Bio Legend | 128016 |
| Ly6C | PerCP-Cy5.5 | HK1.4 | eBioscience | 45-5932-82 |
| Ly6G | PE-Cy7 | RB6-8C5 | eBioscience | 25-5931-81 |
| Ly6G | PE | RB6-8C5 | eBioscience | 12-8931-81 |
| MHC II | Alexa Fluor 700 | I-A/I-E | eBioscience | 56-5321-82 |
| NK1.1 | APC-eFluor 780 | PK136 | eBioscience | 47-5941-80 |

**Supplementary file 1E. Anti-human antibodies used for flow cytometry**

| **Target** | **Fluorochrome** | **Clone** | **Source** | **Catalogue n°** |
| --- | --- | --- | --- | --- |
| CD11c | APC-Cy7 | Bu15 | Bio Legend | 337217 |
| CD14 | PE | 61D3 | eBioscience | 12-0149-71 |
| CD16 | BV570 | 3G8 | Bio Legend | 302035 |
| CD163 | BV421 | GHI/61 | Bio Legend | 333611 |
| CD3 | Alexa Fluor 700 | HIT3a | Bio Legend | 300324 |
| CD19 | Alexa Fluor 700 | HIB19 | Bio Legend | 302225 |
| CD45 | BUV395 | Hi30 | BD Horizon | 563791 |
| CD56 | PE-Cy7 | HCD56 | Bio Legend | 318317 |
| CD66b | APC | G10F5 | Bio Legend | 305117 |
| CD86 | PE-CF594 | 2331 FUN-1 | BD Horizon | 562390 |
| Cx43 | Alexa Fluor 488 | CX-1B1 | Invitrogen | 138388 |

**Supplementary file 1F. Anti-mouse antibodies used for Time-of-Flight Mass Cytometry**

| **Target** | **Clone** | **Atomic mass** | **Element** | **Source** |
| --- | --- | --- | --- | --- |
| Ly6G | 1A8 | 141 | Pr | Fluidigm |
| CD11c | N418 | 142 | Nd | Fluidigm |
| Cx43 | Cx-1B1 | 143 | Nd | Thermo Fisher |
| CD115 | AFS98 | 144 | Nd | Fluidigm |
| F4/80 | BM8 | 146 | Nd | Fluidigm |
| CD45 | 30-F11 | 147 | Sm | Fluidigm |
| CD11b | M1/70 | 148 | Nd | Fluidigm |
| CD19 | 6D5 | 149 | Sm | Fluidigm |
| Ly6C | HK1.4 | 150 | Nd | Fluidigm |
| CD64 | X545/7.1 | 151 | Eu | Fluidigm |
| CD3e | 145-2C11 | 152 | Sm | Fluidigm |
| CD274 | 10F.9G2 | 153 | Eu | Fluidigm |
| CD40 | HM40-3 | 154 | Sm | Bio Legend |
| CD196 | 292L17 | 156 | Gd | Fluidigm |
| CD93 | AA4.1 | 158 | Gd | Fluidigm |
| Gata6 | D61E4 | 161 | Dy | Fluidigm |
| CD1d | 1B1 | 162 | Dy | Fluidigm |
| CD86 | PO3 | 163 | Dy | Bio Legend |
| CD49b/DX5 | HMa2 | 164 | Dy | Fluidigm |
| Arginase-1 | polyclonal | 166 | Er | Fluidigm |
| CX3CR1 | SA011F11 | 167 | Er | Bio Legend |
| CD8a | 53-6.7 | 168 | Er | Fluidigm |
| CD206 | C068C2 | 169 | Tm | Fluidigm |
| CD161 | PK136 | 170 | Er | Fluidigm |
| CD80 | 1610A1 | 171 | Yb | Fluidigm |
| CD4 | RM45 | 172 | Yb | Fluidigm |
| I-A/I-E | M5/114.15.2 | 174 | Yb | Fluidigm |
