# Supplementary material for: Connexin-43-dependent ATP release mediates macrophage activation during sepsis
Source: eLife. 2019 Feb 8;8:e42670. doi: 10.7554/eLife.42670 (PMC6415938; doi:10.7554/eLife.42670)

Source data 1: Full uncut western blot membranes

Figure 2 E

Macrophages isolated from WT

Connexin-43

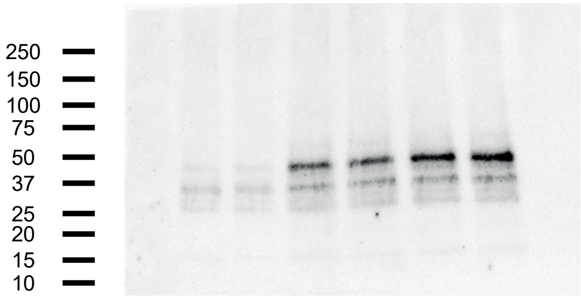

Beta-actin

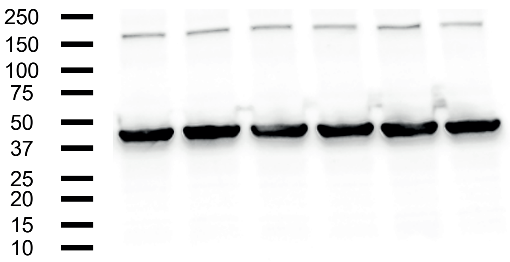

Figure 2 G

Macrophages isolated from MyD88/TRIF KO

Connexin-43

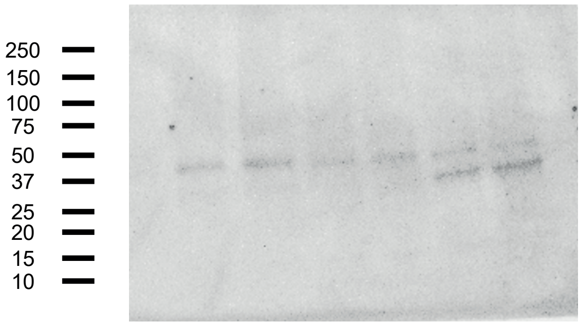

Beta-actin

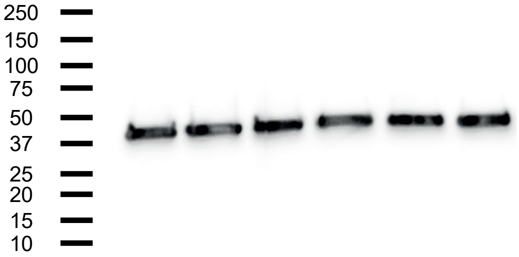

Figure 2 I

Macrophages isolated from Caspase 1/11 KO

Connexin-43

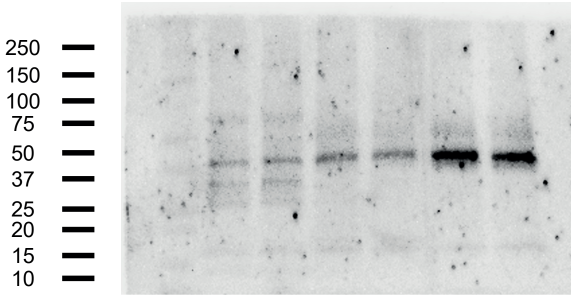

Beta-actin

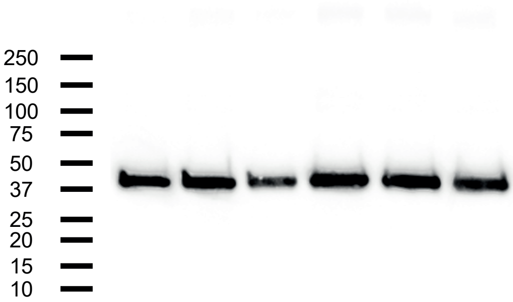

Figure 3 A

Liver - 5 hours after CLP

Connexin-43

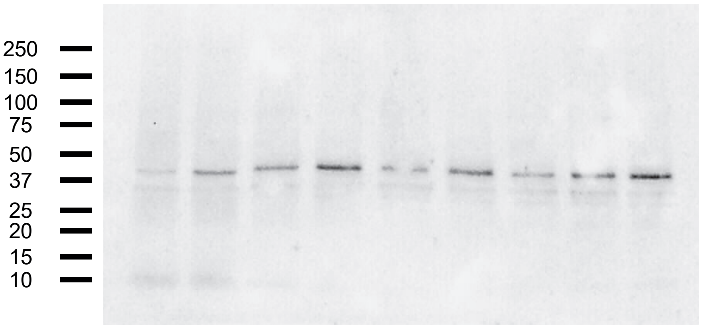

Beta-actin

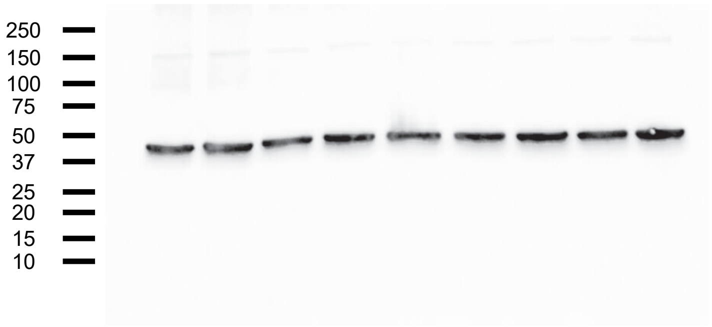

Figure 3 A

Liver - 10 hours after CLP

Connexin-43

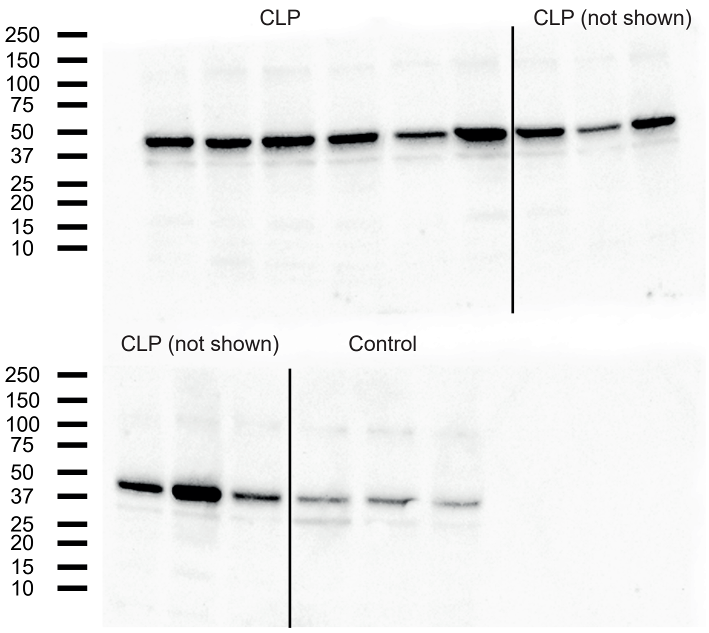

Beta-actin

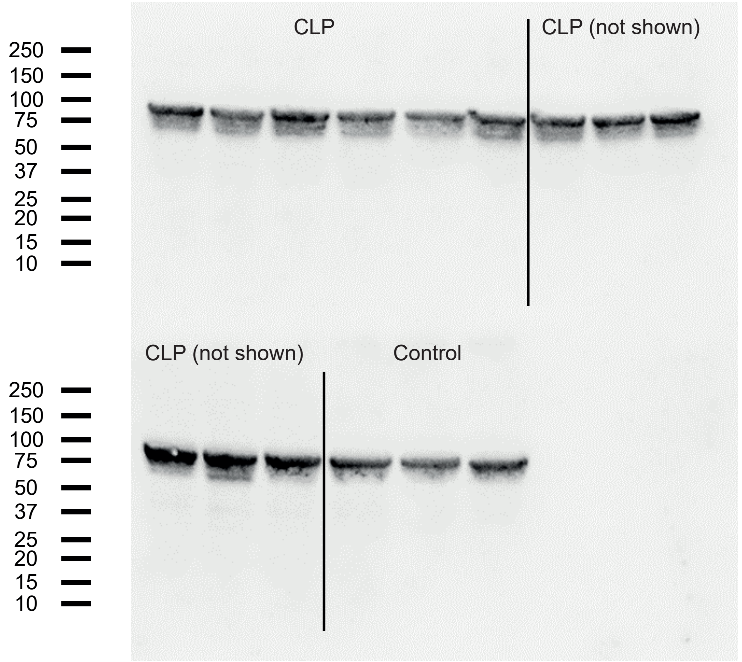

Figure 3 C

Liver - WT versus MyD88/TRIF KO

Connexin-43

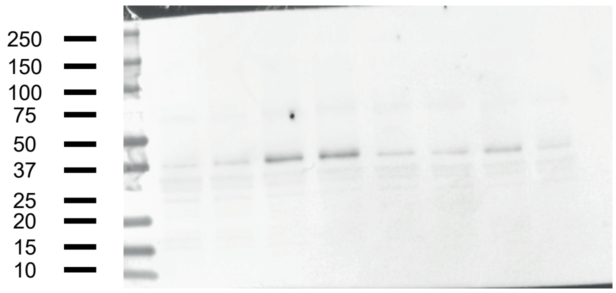

Beta-actin

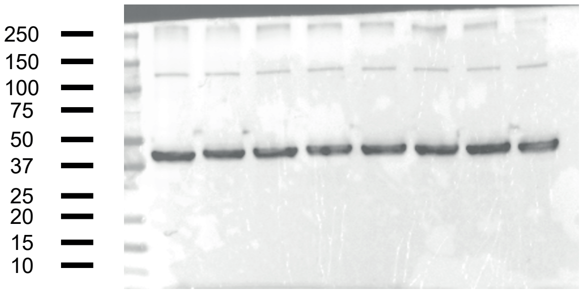

Figure 3 H

Lungs

Connexin-43

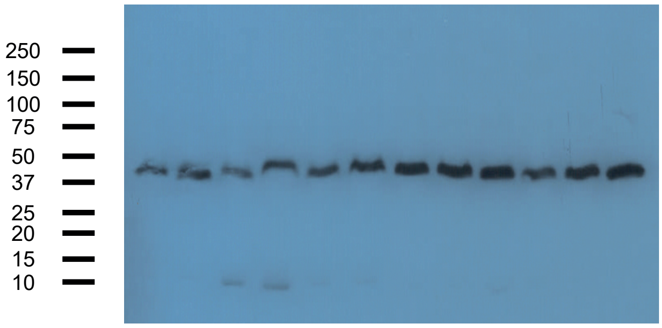

Beta-actin

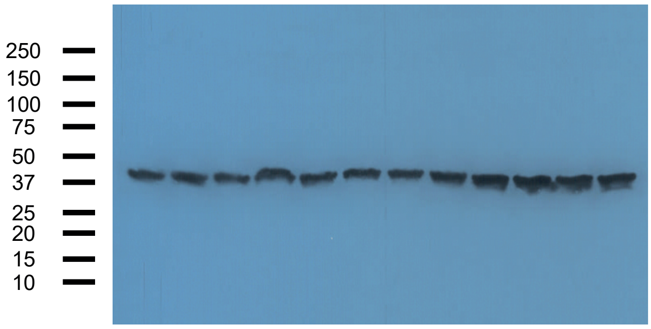

Supplemental Figure 2 C

Macrophages isolated from peritoneal cavity

Connexin-43

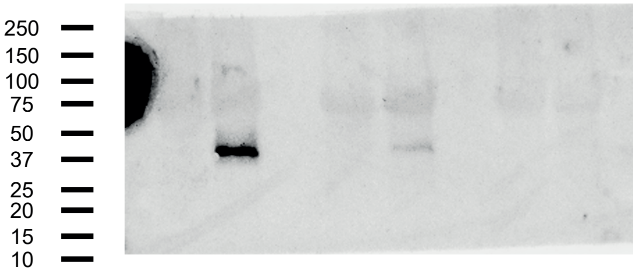

Beta-actin

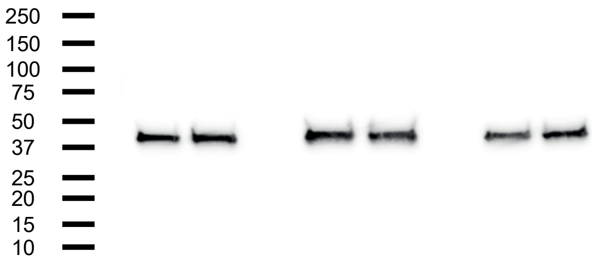

Supplemental Figure 2 D

Liver

Connexin-43

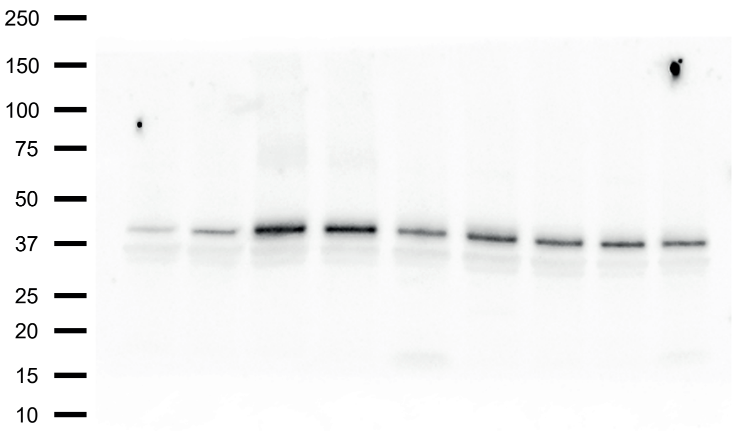

Beta-actin

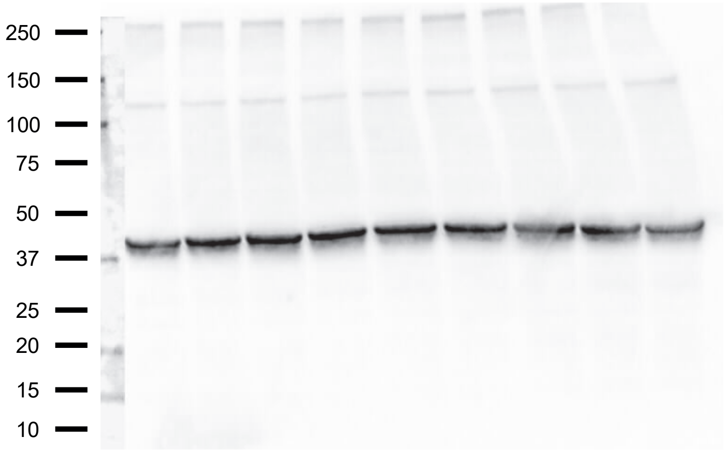

Supplement: Source data 1. [file elife-42670-data1.pdf]
