# Supplementary material for: Connexin-43-dependent ATP release mediates macrophage activation during sepsis
Source: eLife. 2019 Feb 8;8:e42670. doi: 10.7554/eLife.42670 (PMC6415938; doi:10.7554/eLife.42670)
Supplement: Transparent reporting form [file elife-42670-transrepform.pdf]

1st Floor
24 Hills Road
Cambridge CB2 1JP, UK

P 01223 855340
W elifesciences.org
T @elife

eLife's transparent reporting form

We encourage authors to provide detailed information *within their submission* to facilitate the interpretation and replication of experiments. Authors can upload supporting documentation to indicate the use of appropriate reporting guidelines for health-related research (see EQUATOR Network), life science research (see the BioSharing Information Resource), or the ARRIVE guidelines for reporting work involving animal research. Where applicable, authors should refer to any relevant reporting standards documents in this form.

If you have any questions, please consult our Journal Policies and/or contact us: editorial@elifesciences.org.

Sample-size estimation

- You should state whether an appropriate sample size was computed when the study was being designed
- You should state the statistical method of sample size computation and any required assumptions
- If no explicit power analysis was used, you should describe how you decided what sample (replicate) size (number) to use

Please outline where this information can be found within the submission (e.g., sections or figure legends), or explain why this information doesn't apply to your submission:

Sample size calculation was performed prior to experiments on ClinCalc.com and using statistical methods previously published (1) (please see material and methods). The variability in the outcome of sepsis in caecal ligation and puncture model was expected to be high. Briefly, sample size calculation (significance 5%, power 80%) revealed that in order to detect a significant difference of 25% (increase or decrease) in cytokines and ATP measurements, a number of at least 8 animals per group would be necessary. For survival experiments, survival times between 24 hours and up to one week for high grade sepsis in wild type animals were expected (2). A sample size calculation (significance 5%, power 80%) revealed group numbers of 10 animals per group in case survival time would be doubled in Gap-27 treated animals or Connexin-43 conditional KO animals compared to non-treated wild-type animals. Papers publishing results of survival experiments in sepsis field confirm these numbers (3).

No explicit sample size calculation was performed prior to in vitro experiments. A number of at least three biological replicates was used for each experiment in order to allow statistical tests to be performed.

(1) Rosner B, Fundamentals of Biostatistics, 2011.
(2) Rittirsch D et al., Nat. Protoc., 4(1), 31 (2009).
(3) Csoka B et al., FASEB J., 29(9), 3626 (2015).

Replicates

- You should report how often each experiment was performed
- You should include a definition of biological versus technical replication
- The data obtained should be provided and sufficient information should be provided to indicate the number of independent biological and/or technical replicates
- If you encountered any outliers, you should describe how these were handled
- Criteria for exclusion/inclusion of data should be clearly stated

- High-throughput sequence data should be uploaded before submission, with a private link for reviewers provided (these are available from both GEO and ArrayExpress)

Please outline where this information can be found within the submission (e.g., sections or figure legends), or explain why this information doesn't apply to your submission:

We performed each experiment and confirmed our results at least three times before inclusion in the manuscript. In vitro experiments, in particular experiments investigating ATP release, were repeated up to five times.

Information regarding the nature of replicates (biological vs. technical) is indicated in the figure legend for each experiment.

Raw data and statistical tests are available for consideration in a separate PRISM file, that we would be happy to send you.

As expected with our sepsis model, we encountered some outliers in our results. In Figure 4 D, we removed 7 outliers after performing a ROUT test (Q = 1%) using Graphad PRISM 6 software. This is indicated in the figure legend. No outliers were removed in other experiments.

Statistical reporting

- Statistical analysis methods should be described and justified
- Raw data should be presented in figures whenever informative to do so (typically when N per group is less than 10)
- For each experiment, you should identify the statistical tests used, exact values of N, definitions of center, methods of multiple test correction, and dispersion and precision measures (e.g., mean, median, SD, SEM, confidence intervals; and, for the major substantive results, a measure of effect size (e.g., Pearson's r, Cohen's d)
- Report exact p-values wherever possible alongside the summary statistics and 95% confidence intervals. These should be reported for all key questions and not only when the p-value is less than 0.05.

Please outline where this information can be found within the submission (e.g., sections or figure legends), or explain why this information doesn't apply to your submission:

The way we performed statistical analyses are detailed and justified in a specific section of the material and methods (please see Statistical analysis, page 27). In addition, statistical tests used are detailed for each experiment in the corresponding figure legend. P-values are symbolized on the figure using stars and corresponding legend can be found in the material and methods.

We specify N for each experiment in the figure legends. When ANOVA and multiple comparisons were performed, a statistical correction (Turkey) of the p-value for multiple comparison was applied.

(For large datasets, or papers with a very large number of statistical tests, you may upload a single table file with tests, Ns, etc., with reference to sections in the manuscript.)

Group allocation

- Indicate how samples were allocated into experimental groups (in the case of clinical studies, please specify allocation to treatment method); if randomization was used, please also state if restricted randomization was applied
- Indicate if masking was used during group allocation, data collection and/or data analysis

Please outline where this information can be found within the submission (e.g., sections or figure legends), or explain why this information doesn't apply to your submission:

For human samples, allocation to one group (control versus peritonitis) was decided according to the diagnosis, that are detailed for each patient in supplemental table 1.

Mice were allocated to different groups according to the treatment they received. Experimental settings are detailed in the material and methods. Animal experiments were planned, carried out and reported in agreement with current 3R and ARRIVE guidelines.

Additional data files ("source data")

- We encourage you to upload relevant additional data files, such as numerical data that are represented as a graph in a figure, or as a summary table
- Where provided, these should be in the most useful format, and they can be uploaded as "Source data" files linked to a main figure or table
- Include model definition files including the full list of parameters used
- Include code used for data analysis (e.g., R, MatLab)
- Avoid stating that data files are "available upon request"

Please indicate the figures or tables for which source data files have been provided:

Western blots – full uncut gels
Raw data – PRISM file

eLife Sciences Publications, Ltd is a limited liability non-profit non-stock corporation incorporated in the State of Delaware, USA, with company number 5030732, and is registered in the UK with company number FC030576 and branch number BR015634 at the address 1st Floor, 24 Hills Road, Cambridge CB2 1JP | August 2014
